# An In Silico and In Vitro Approach Identified Potential Trypanothione Synthetase Inhibitors with Trypanocidal Activity

**DOI:** 10.3390/molecules31071139

**Published:** 2026-03-30

**Authors:** Rogelio Gómez-Escobedo, Domingo Méndez-Álvarez, Alma D. Paz-González, Eyra Ortiz-Pérez, Lenci K. Vázquez-Jiménez, Ana Verónica Martínez-Vázquez, Timoteo Delgado-Maldonado, José M. Quintero-Solano, Citlali Vázquez, Emma Saavedra, Guadalupe Avalos-Navarro, Karina Vázquez, Gildardo Rivera, Benjamín Nogueda-Torres

**Affiliations:** 1 Departamento de Parasitología, Escuela Nacional de Ciencias Biológicas, Instituto Politécnico Nacional, Ciudad de México 11340, Mexico; rogelio.gomez.14@gmail.com; 2 Laboratorio de Biotecnología Farmacéutica, Centro de Biotecnología Genómica, Instituto Politécnico Nacional, Reynosa 88710, Mexico; doomadv@hotmail.com (D.M.-Á.); apazg@ipn.mx (A.D.P.-G.); eortizp@ipn.mx (E.O.-P.); lenka.18@hotmail.com (L.K.V.-J.); avmartinez@ipn.mx (A.V.M.-V.); titi_999@live.com (T.D.-M.); jquinteros2300@alumno.ipn.mx (J.M.Q.-S.); 3 Departamento de Bioquímica, Instituto Nacional de Cardiología Ignacio Chávez, Ciudad de México 14080, Mexico; cita2812@gmail.com (C.V.); emma_saavedra2002@yahoo.com (E.S.); 4 Departamento de Ciencias Médicas y de la Vida, Centro Universitario de la Ciénega (CUCIÉNEGA), Universidad de Guadalajara, Av. Universidad 1115, Lindavista, Ocotlán 47820, Mexico; guadalupe.avalos5337@academicos.udg.mx; 5 Facultad de Medicina Veterinaria y Zootecnia, Universidad Autónoma de Nuevo León, C. Francisco Villa 20, Hacienda del Cañada, General Escobedo 66054, Mexico; karina.vazquezcn@uanl.edu.mx

**Keywords:** Chagas disease, trypanothione synthetase, molecular docking, repositioning, FDA drugs

## Abstract

In this study, a drug repurposing strategy was implemented with the aim of identifying new trypanocidal agents against *Trypanosoma cruzi* (*T. cruzi*). A total of 924 Food and Drug Administration (FDA)-approved drugs were screened by molecular docking on three sites of trypanothione synthetase (TS), including the catalytic site, a blind docking site, and a potential allosteric site. Selected compounds were further evaluated through in vitro and in vivo assays. Tadalafil, Zafirlukast, Raltegravir, and Olmesartan had better trypanocidal activity than the reference drugs Benznidazole and Nifurtimox in the in vitro evaluation against the trypomastigote form. Additionally, these drugs were able to decrease parasitemia by 20–50% in mice in an acute treatment. Molecular dynamics simulations (MDS) at 120 ns helped link findings from in vitro/in vivo experiments to a potential mechanism of action targeting *T. cruzi* trypanothione synthetase (*Tc*TS). Therefore, the results encourage the use of these drugs to develop new anti-*T. cruzi* agents.

## 1. Introduction

Chagas disease is an anthropozoonosis caused by the infection with the protozoan parasite *Trypanosoma cruzi* (*T. cruzi*), with 6 to 7 million people affected, mainly in Latin America and the Caribbean [1,2]; however, due to large migrations and climate change, the disease is found in non-endemic regions such as the United States, Canada, Europe, Japan, and Australia [1,3]. This disease includes a wide spectrum of clinical manifestations, highlighted in the chronic phase; cardiac affectations such as left ventricular diastolic dysfunction, dilated cardiomyopathy, arrhythmias, thromboembolic events, and terminal heart failure; and gastrointestinal manifestations such as megaesophagus and megacolon [4].

Although Chagas disease was medically described over 100 years ago, there remains a need to develop new pharmacological treatments. The drugs currently available for its treatment are Nifurtimox (Nfx) and Benznidazole (Bzn), two nitroaromatic compounds that have been in use since the late 1960s and early 1970s, respectively, both showing acceptable effectiveness in the acute phase. However, their use has severe adverse effects, including gastrointestinal disturbances, neurological symptoms, and hypersensitivity reactions, which cause people to abandon the treatment, resulting in a lack of parasitological cure and creation of drug-resistant strains. Additionally, it has been proven that both drugs have a low percentage of parasitological cure (8–20%) in the chronic phase of the disease. Although both drugs are used clinically, Bzn is considered the first-line treatment in most endemic countries due to its greater implementation in clinical practice [5].

Several strategies have been applied to find new options for Chagas disease pharmacological treatment, for example, the chemical synthesis of new compounds or searching for complex mixtures of natural compounds with trypanocidal activity. These processes require long periods of basic and clinical research, with the concomitant higher costs, before obtaining a drug with the desired antiparasitic effect. Currently, drug repositioning is one of the main strategies to identify new pharmacological options for various diseases in a faster and more cost-effective manner, as it circumvents much of the time required for toxicological evaluation in humans. This approach has been successfully applied in diseases such as malaria and tuberculosis [6,7], and therefore represents a promising strategy to identify new antichagasic compounds. In addition to their individual activity, the potential use of these compounds in combination with existing therapies, such as Bzn, could be explored in future studies. Drug combination strategies have shown beneficial effects in other infectious diseases, including improved efficacy and reduced toxicity [8,9]; however, this approach was beyond the scope of the present study.

Different pharmacological targets have been studied in *T. cruzi*; among them are ergosterol synthesis [10], glycolysis [11], the purine rescue pathway [12], DNA topoisomerases [13], folate metabolism [14], polyamines [15], and antioxidant metabolism [16]. The latter is of particular interest because it is a distinctive and unique characteristic of *T. cruzi*, since it is based on trypanothione, which is synthesized by the enzyme trypanothione synthetase (TS), which is absent in human cells. TS catalyzes the conjugation of two molecules of glutathione to the terminal amino groups of spermidine. TS is a cytosolic monomer with two catalytic domains; the C-terminal is the synthetase domain where trypanothione is synthesized, whereas the N-terminal is a histidine-dependent aminohydrolase amidase domain. In this study, we will only focus on the synthetase domain, which has an ATP-grabbing folding, with the catalytic site being a triangular-shaped cavity into which the three necessary substrates (ATP, spermidine or glutathionyl-spermidine, and glutathione) are coupled to obtain trypanothione [17]. Therefore, TS is considered a pharmacological target, since its inhibition can decrease the trypanothione pool, thus affecting its trypanothione-dependent antioxidant defense, compromising its survival during the infection of the host cell. To date, few studies have been carried out on this enzyme.

In this study, using the *T. cruzi* TS (*Tc*TS) enzyme as a pharmacological target, an in silico molecular docking screening of FDA-approved drugs was performed on three sites of the enzyme: (a) at the catalytic site; (b) at a site determined by a blind molecular docking analysis; and (c) at a potential allosteric site. Since the experimental three-dimensional structure of *Tc*TS has not been elucidated by physical methods, a homology model was constructed and used for all in silico analyses.

Instead of pre-screening compounds based on previously described pharmacological activities, an unbiased virtual screening approach was implemented, which included a total of 924 FDA-approved drugs. This strategy was designed to explore a broad chemical spectrum and identify potential *Tc*TS inhibitors beyond those with known antioxidant or antiparasitic properties. In this regard, molecular docking was employed as a high-throughput filtering tool to detect non-obvious interactions and prioritize candidates for further biological evaluation. This workflow follows a widely used strategy in drug repurposing studies; this approach enables a more efficient use of resources while maintaining the possibility of identifying novel bioactive compounds.

In addition, in vitro and in vivo biological evaluations of the selected FDA-approved drugs were performed against blood trypomastigotes of NINOA and INC-5 strains. Bzn was selected as the reference drug in the in vivo trials, while Nfx was included as a comparator drug in the corresponding in vitro analyses.

## 2. Results

### 2.1. Modeling of T. cruzi Trypanothione Synthetase

**Figure 1:**
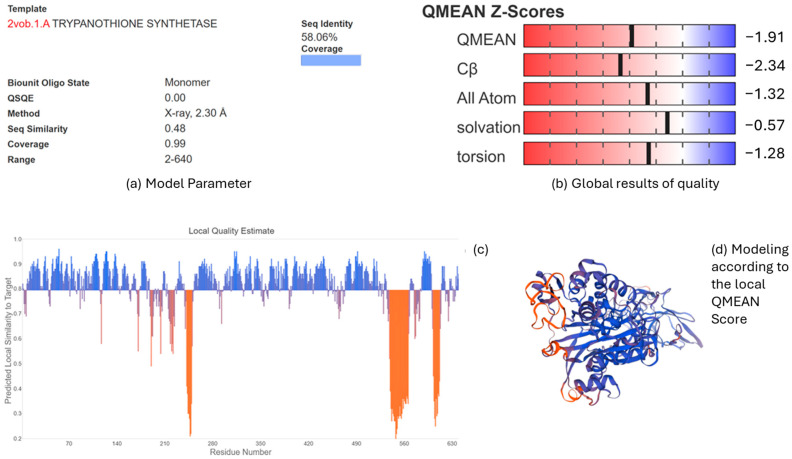
Parameters of validation of the *Tc*TS three-dimensional model: (**a**) model parameters; (**b**) global results of quality, where red indicates low quality, white intermediate quality, and blue high-quality regions; (**c**) Local quality estimation along the protein sequence, where blue regions represent high-confidence residues and orange/red regions indicate lower confidence; and (**d**) results of the local quality assessment, where blue denotes high structural reliability and orange indicates regions of lower confidence.

Since the experimental three-dimensional structure of *Tc*TS is not currently available, a homology model was constructed for this study, as mentioned above. The model was generated using the amino acid sequence obtained from the NCBI protein database (accession number AAG15409.1, CL Brener strain) and built with the SwissModel software (https://swissmodel.expasy.org/). The model parameters, global result quality, local quality estimate, and QMEAN score are shown in Figure 1.

**Figure 2:**
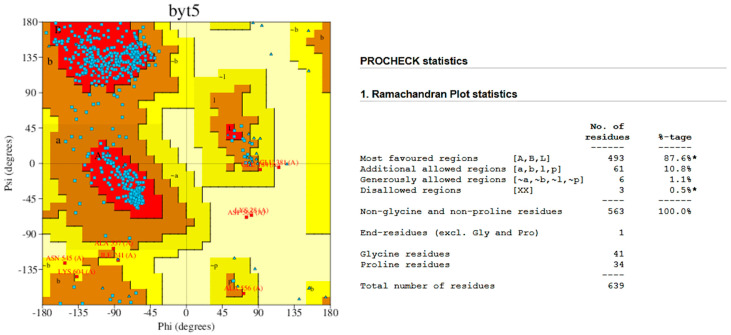
Ramachandran plot of *Tc*TS modeling. * indicate values interpreted according to reference distributions from high-quality protein structures, as defined by PROCHECK in the Ramachandran plot analysis. The clustering of residues reflects the high density of conformations within favored regions.

The Ramachandran plot (Figure 2) of the *Tc*TS three-dimensional model, obtained using the ProCheck software (https://www.ebi.ac.uk/thornton-srv/software/PROCHECK/, accessed on 30 November 2025) [18], showed 87.6% of positions in the favorable regions and 10.8% in the allowed regions.

### 2.2. Molecular Docking of Control Inhibitors on TcTS

**Figure 3:**
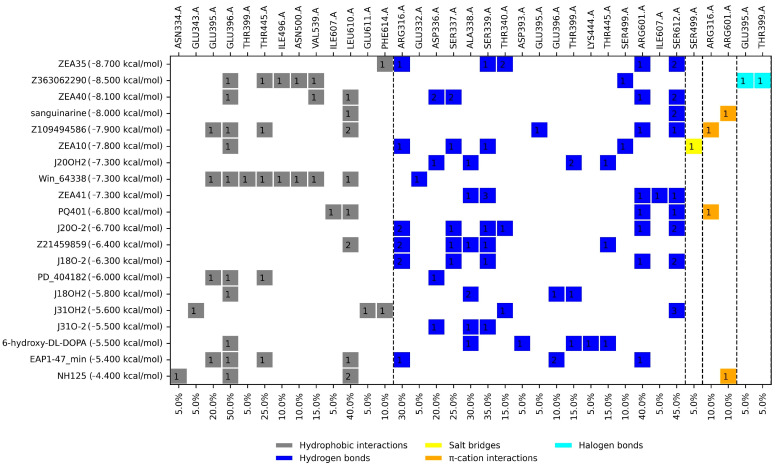
Docking score and profile ligand–protein interactions for the control inhibitors docked on the *Tc*TS catalytic site. Dotted lines separate the types of interactions. The figure inside the boxes indicates the number of interactions with the ligand.

A molecular docking analysis of 20 *Tc*TS inhibitors (ZEA35, Z363062290, ZEA40, ZEA41, ZEA10, Z109494586, J20OH2, PQ401, J2O-2, Z21459859, J18O-2, PD_404182, J18OH2, J31OH2, J31O-2, EAP1-47, NH125, Win_64338, 6-hydroxy-DL-DOPA, and sanguinarine) [19,20] was carried out on the catalytic site. The docking score and profile interactions are shown in Figure 3.

**Figure 4:**
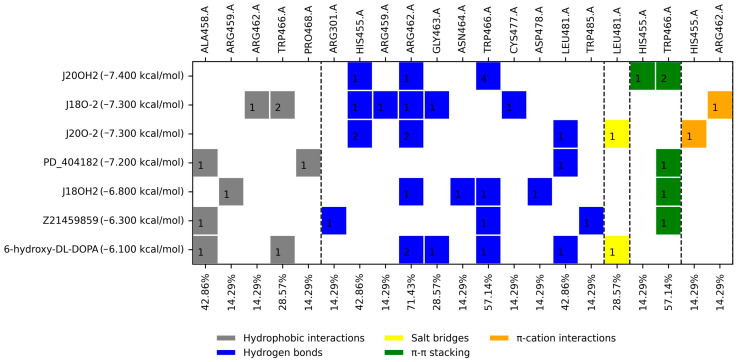
Docking score and profile ligand–protein interactions for the control inhibitors docked on the *Tc*TS potential allosteric site. Dotted lines separate the types of interactions. The figure inside the boxes indicates the number of interactions with the ligand.

Additionally, a blind molecular docking analysis of the control inhibitors bound to *Tc*TS was performed to identify additional potential binding sites (Figure 4). The ligands 6-hydroxyl-DL-DOPA, PD_404182, Z21459859, J18O-2, J18OH2, J20O-2, and J20OH2 bound at the same site (X = −27.871, Y = −0.318, and Z = −17.397); therefore, this region was also considered a potential (allosteric) binding site for molecular docking analysis of the FDA drugs.

### 2.3. Screening of FDA-Approved Drugs by Molecular Docking

Based on the drug repurposing strategy described above, a total of 924 FDA-approved drugs were screened by molecular docking on the catalytic site and potential allosteric sites of *Tc*TS. Additionally, blind molecular docking of these compounds was performed to identify additional potential binding regions. The results of the top 20 FDA-approved drugs at each site, according to docking score and ligand–protein interaction profiles, are presented in Table 1.

#### 2.3.1. Molecular Docking of FDA-Approved Drugs on the Catalytic Site

**Figure 5:**
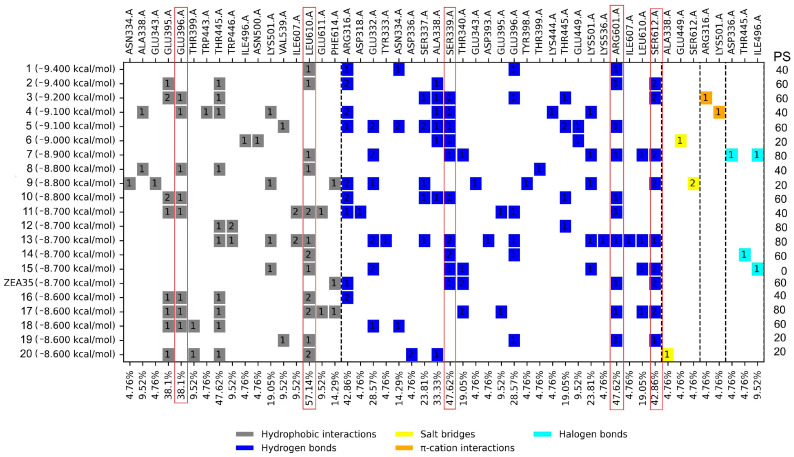
Docking score and profile ligand–protein interactions for the top 20 FDA drugs and the control inhibitor docked on the *Tc*TS catalytic site. PS: Percentage of similarity. Red boxes mark the five amino acids that are part of the consensus profile in the docked inhibitors. Dotted lines separate the types of interactions. The figure inside the boxes indicates the number of interactions with the ligand.

The molecular docking analysis of the 924 FDA-approved drugs on the catalytic site enables the selection of the top 20 compounds (Figure 5) with docking score ranging from −8.6 to −9.4 kcal/mol. The control inhibitor ZEA35 had a docking score of −8.7 kcal/mol. Next, ligand–protein interaction profiling of the top 20 FDA drugs and the control was performed. Figure 5 shows the profile interaction with hydrophobic interactions (HI), hydrogen bonds (HB), π–cation interactions (π-C), halogenated interactions (HalB), and salt bridges (SB).

#### 2.3.2. Molecular Docking of FDA-Approved Drugs on the Probable Allosteric Site

**Figure 6:**
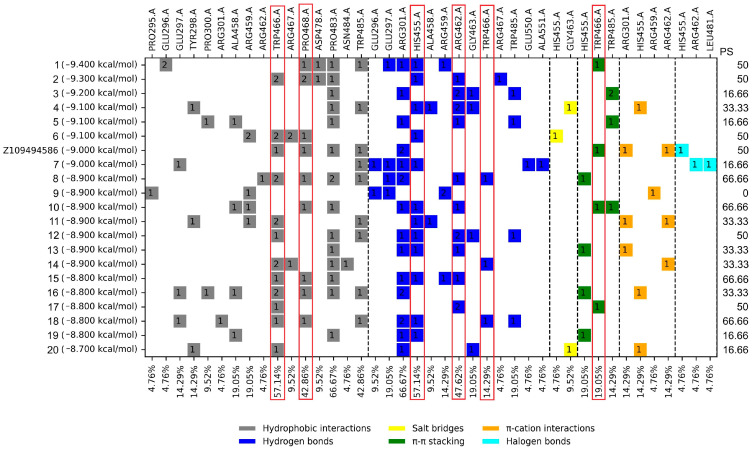
Docking score and profile of ligand–protein interactions for the top 20 FDA drugs and the ligand inhibitor control docked at the putative allosteric site. PS: Percentage of similarity. Red boxes mark the six amino acids that are part of the consensus profile in the docked inhibitors. The dotted lines separate the types of interactions. The figure inside the boxes indicates the number of interactions with the ligand.

In a blind molecular docking, several control inhibitors were observed to bind at a particular site of *Tc*TS different from the catalytic one, suggesting a putative allosteric site. Therefore, molecular docking analysis of the FDA drugs at that site was performed (Table 1). The top 20 compounds showed a docking score of −8.7 to −9.4 kcal/mol, whereas the control inhibitor Z109494586 displayed a docking score of −9.0 kcal/mol. Figure 6 shows the profile of ligand–protein interactions.

#### 2.3.3. Blind Molecular Docking of FDA-Approved Drugs on *Tc*TS

**Figure 7:**
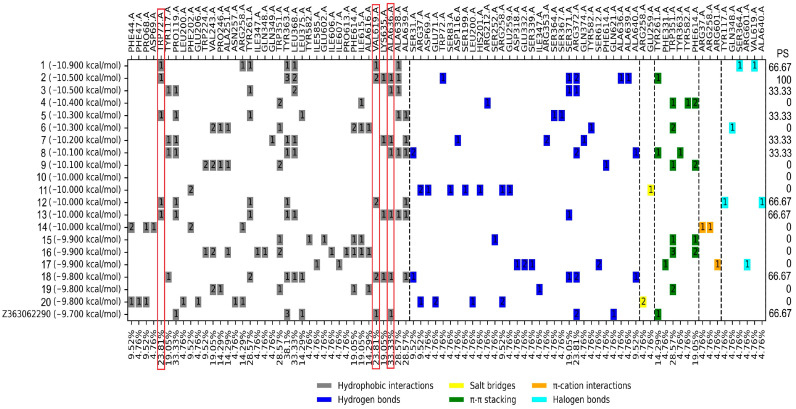
Docking score and profile ligand–protein interaction for the top 20 FDA drugs and their controls based on blind molecular docking analysis. PS: Percentage of similarity. Red boxes mark the three amino acids that are part of the consensus profile in the docked inhibitors. Dotted lines separate the types of interactions. The figures inside the boxes indicate the number of interactions with the ligand.

A blind molecular docking of the 924 drugs on the *Tc*TS enzyme was performed. The results of the top 20 compounds are shown in Table 1, where the docking score ranged from −9.8 to −10.9 kcal/mol, which is better than the control Z363062290 with −9.7 kcal/mol. The analysis of the protein–ligand interaction profile is shown in Figure 7.

**Figure 8:**
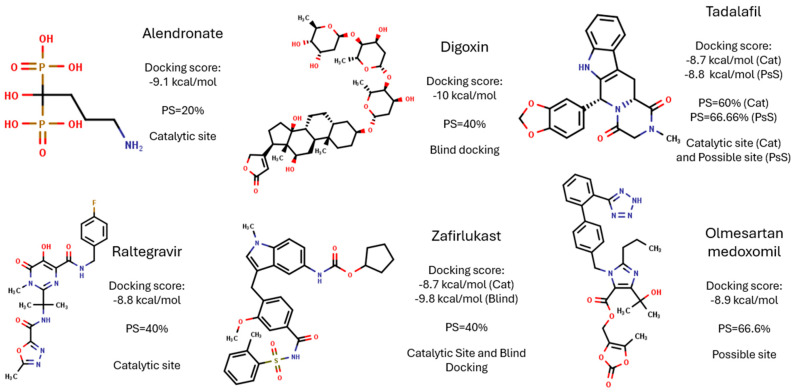
FDA drugs selected for biological evaluation against *T. cruzi* blood trypomastigotes in an in vitro model.

After molecular docking analyses, from the top 20 drugs with the highest binding docking score and interaction profiles, some drugs were selected for further studies. The following criteria were applied: (1) easy availability, access, and storage; (2) oral administration; and (3) that the described adverse effects are common and less severe than those of the reference drugs, Nfx and Bzn. Six drugs were selected to continue with their biological evaluation (Figure 8), although in this work, we only show the results of Tadalafil, Raltegravir, Zafirlukast, and Olmesartan, as the biological activity of Digoxin and Alendronate sodium has been previously reported by our research group in the context of a different molecular target in *T. cruzi* [21]. Therefore, to avoid redundancy and maintain the focus of the present study on *Tc*TS, these results are not included.

### 2.4. In Vitro Activity Against Blood Trypomastigotes

The four FDA drugs selected were evaluated at a single concentration to determine their trypanocidal activity against blood trypomastigotes compared with the reference drugs (Table 2). All compounds showed similar lysis percentages to the reference drugs Nfx and Bzn; therefore, the half-maximal lethal concentration (LC_50_) of all drugs was calculated for both strains. Tadalafil, Zafirlukast, Raltegravir, and Olmesartan showed lower LC_50_ values (between 30 and 177 µM) than the reference drugs (LC_50_ between 161 and 337 µM) in both strains, with Zafirlukast and Olmesartan standing out with LC_50_ values of 32 and 78 µM, respectively, against the NINOA strain and 30 and 52 µM, respectively, against the INC-5 strain (Table 2). Therefore, acute treatment studies were conducted via in vivo monotherapy trials in infected mice.

### 2.5. In Vivo Evaluation of Acute Monotherapy in a Murine Model of T. cruzi Infection

**Figure 9:**
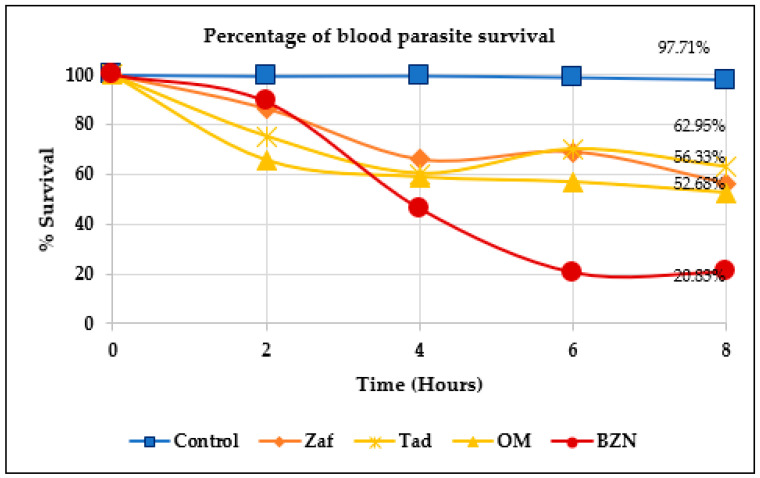
Percentage of blood parasite survival elicited by three selected FDA drugs and Benznidazole in acute treatment in an animal mouse model of infection with *T. cruzi* NINOA strain. Zaf: Zafirlukast; Tad: Tadalafil; OM: Olmesartan; BZN: Benznidazole.

Results corresponding to the evaluation of Tadalafil, Zafirlukast, and Olmesartan (Raltegravir was not evaluated) in an acute in vivo model of infection with the NINOA strain in mice are shown in Figure 9.

### 2.6. Molecular Dynamics Simulations (MDS)

**Figure 10:**
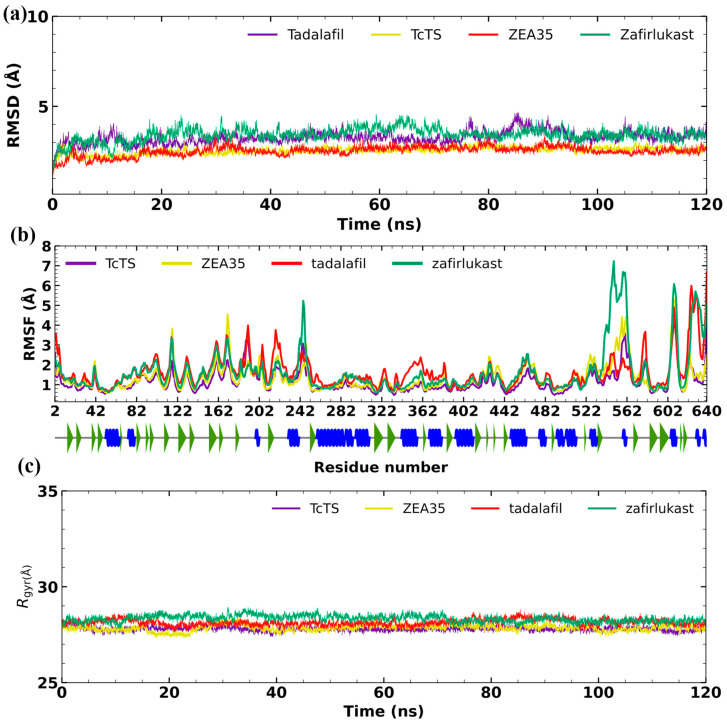
Molecular dynamics studies on the catalytic site of *Tc*TS. (**a**) Graph of the global RMSD values of the simulation, (**b**) graph of the global RMSF values of the simulation (the alpha helix is shown as a spiral blue line, the gray line corresponds to the loops, and the green arrow represents beta-sheets), and (**c**) graph of the global radius of gyration values of the simulation.

Based on the in vivo results, molecular dynamics (MD) simulations were performed to help explain the behavior of the protein–ligand complex in a biological system. Hence, zafirlukast, tadalafil, and the reference inhibitor ZEA35 were subjected to 120 ns of simulation on the catalytic site (Figure 10). As a first step, the apo form of the protein was simulated at 120 ns. This was to evaluate the stability of the molecule in the prepared system. The protein showed an average RMSD of 2.5 ± 0.1 Å, indicating stability. These results also suggest that the *Tc*TS model generated is stable and suitable for the in silico studies. Inhibitor ZEA35 in complex with *Tc*TS showed an RMSD value of 2.5 ± 0.2 Å, which is consistent with previously reported as an inhibitor in in vitro assays. Therefore, these values are considered stable under the conditions evaluated. Zafirlukast (RMSD = 3.4 ± 0.3 Å) and tadalafil (RMSD = 3.2 ± 0.3 Å) in complex with *Tc*TS at the catalytic pocket evidenced RMSD values less than 4 Å. Likewise, the RMSF values were less than 1.8 Å in all cases. Finally, the radius of gyration values for the *Tc*TS (apo form) were 23.9 ± 3.0 Å. Similar findings were observed for the complexes.

**Figure 11:**
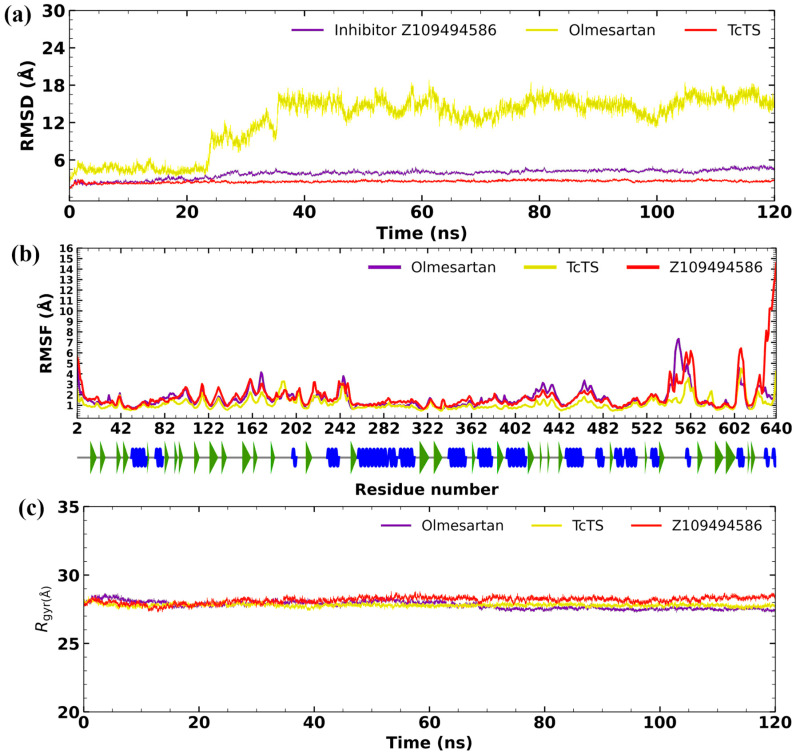
Molecular dynamics studies in the allosteric site of *Tc*TS. (**a**) Graph of the RMSD values of the simulation, (**b**) graph of the RMSF values of the simulation (the alpha helix is shown as a spiral blue line, the gray line corresponds to the loops, and the green arrow represents beta-sheets), and (**c**) graph of the radius of gyration values of the simulation.

Olmesartan and the inhibitor Z109494586 were evaluated in the predicted allosteric site (Figure 11). The allosteric inhibitor Z109494586 had an RMSD value of 1.5 ± 0.5 Å throughout the simulation. Meanwhile, olmesartan exhibited an RMSD value of 3.4 ± 0.3 Å. On the other hand, the RMSF values were less than 1.8 Å in all cases. Lastly, the radius of gyration values were shown to be similar than found for the free form of *Tc*TS.

## 3. Discussion

Molecular modeling of the *Tc*TS protein was performed since its crystal structure has not been determined. The highest degree of structural identity was observed with the crystallized TS enzyme from *L. major* (PDB: 2VOB), with a sequence identity of 58.6% and a coverage of 99%. In the three-dimensional model (Figure 1), regions lacking quality were located at the periphery of the enzyme (orange color), far from the catalytic site. The Ramachandran plot showed the percentage of amino acids in the favorable regions and in the allowed areas, suggesting a good quality of the *Tc*TS protein structure. Fyfe and coworkers carried out an overlay analysis of the TS of *L. major* and glutathionyl spermidine synthetase amidase (enzyme homolog) from *E. coli* to determine the glutathione binding site in the synthetase domain [22]. Their results showed a cavity formed by the amino acid residues Arg328, Ser349, Ser351, Asp403, Glu407, Glu408, Met459, and Ser462. Therefore, to identify the corresponding residues in the *Tc*TS model, a PDB-fold analysis was performed. The catalytic site corresponded to amino acids Arg316, Ser337, Ser339, Asp391, Glu395, Glu396, Met447, and Thr450, establishing the coordinates for the molecular docking analysis (X = −11.729, Y = −10.560, and Z = −2.080).

### 3.1. Molecular Docking on the Catalytic Site

To select the FDA-approved drugs as potential inhibitors of *Tc*TS, a comparison was made with the protein–ligand interaction profile of the control inhibitors. For the catalytic site, the five most frequent interactions were determined: HI: Glu396 and Leu610; HB: Ser339, Arg601, and Ser612. Of the top 20 FDA drugs, only eight did not meet the best docking score and percent similarity (≥40%) of ligand–protein interaction (right side of Figure 5). Additionally, other criteria were used to rule out drugs from further analyses: among them, those whose molecular weight was ≥1000 g/mol that could come to be considered as pan-assay interference compounds (PAINS), since they tend to interact nonspecifically with many pharmacological targets, as well as those that caused similar or worse adverse effects than Nfx and Bzn, among other criteria mentioned above. Therefore, regarding the catalytic site docking results, the drugs Raltegravir, Zafirlukast, and Tadalafil were selected.

Molecular docking results suggest that Tadalafil, Raltegravir, and Zafirlukast exhibit distinct binding profiles on the *Tc*TS catalytic site that are comparable to previously reported inhibitors [19,20], which is relevant since, as previously mentioned, this enzyme is absent in mammalian hosts, making it an attractive target. Furthermore, the recurring interactions with residues Glu396 and Ser339 are of particular interest because they could contribute to a stable interaction capable of interfering with the enzyme’s synthetic function. These findings therefore support the proposal that these FDA-approved drugs exert a trypanocidal effect at least by interfering with trypanothione synthesis.

### 3.2. Molecular Docking on the Potential Allosteric Site

As previously mentioned, when blind molecular docking was performed, it was observed that several controls (6-hydroxyl-DL-DOPA, PD_404182, Z21459859, J18O-2, J18OH2, J20O-2, and J20OH2) bound at a specific site with coordinates X = −27.871, Y = −0.318, and Z = −17.397. For this case, the interaction profile of the control ligands was analyzed, and six amino acids with the highest percentage of occurrence were found: HI: Trp466 and Pro468; HB: His455, Arg462, and Trp466; and π-π: Trp466. The first four were the most recurrent in terms of FDA drugs. The drugs with the best docking score and PS ≥ 40% were Itraconazole, Ergotamine, Adapalene, Oxytetracycline, Paliperidone, Olmesartan, Rifaximin, Tadalafil, and Oxymorphone. Considering the previous selection criteria, only Olmesartan and Tadalafil were selected for further assays. It is interesting to consider Olmesartan, an angiotensin II receptor antagonist that is used for the treatment of cardiovascular diseases; although it is used to treat hypertension in the first place, it is striking that a drug was presented for this type of disease, considering that Chagas disease, in its chronic stage, causes damage to the heart, generating Chronic Chagas cardiomyopathy. There are few reports of allosteric inhibition in *T. cruzi* enzymes, and much less in *Tc*TS, thus highlighting the importance and originality of this finding; however, the need for validation through experiments arises.

### 3.3. Blind Molecular Docking

A ligand–protein profile of the top 20 FDA drugs was analyzed by blind molecular docking on *Tc*TS, and a comparison with the profile of the controls used was carried out. It is important to mention that being a blind molecular docking, the frequency of amino acids with respect to controls was very low; therefore, those with a percentage of occurrence of 20–30% were considered: HI: Trp72, Tyr363, and Val619; HB: Arg462; and π-π: Trp466. Figure 4 shows that only the HIs mentioned appear in the ligand–protein profile of the FDA-approved drugs, with five drugs having a similarity percentage of 66.66%: Dutasteride, Tripranavir, Digoxin, Conivaptan, and Zafirlukast. Applying the previous criteria, only Zafirlukast was selected for further assays.

Blind docking showed Zafirlikast as one of the most consistent ligands, even when compared to control compounds. It is noteworthy to mention that this drug has been previously described as a drug with several molecular targets, giving it the possibility of facing the parasite on different fronts; this is an advantageous feature, since polypharmacology has been considered as a strategy to avoid the selection of resistant strains. However, it is important to consider the interactions that these could have outside the target. Zafirlikast meets the criteria mentioned above, which is why it was chosen to be able to continue with the biological evaluations.

### 3.4. In Vitro Activity on Blood Trypomastigotes

Drug evaluation against the trypomastigote stage is of great relevance since it is the parasite form responsible for the acute phase of Chagas disease. Table 2 shows the results of the in vitro trypanocidal activity of the FDA and reference drugs at a single concentration as well as the LC_50_ values from dose-dependent assays. At 12.5 µg/mL, the four FDA drugs against both strains (NINOA and INC-5) had a percentage of lysis similar to or equal to the reference drugs. Also, all the FDA drugs presented lower or similar LC_50_ values than the reference drugs. Tadalafil and Raltegravir were less potent, and Zafirlukast was the most active. Therefore, studies in acute treatment in in vivo assays in infected mice in monotherapy were performed.

### 3.5. Short-Term In Vivo Trial of Monotherapy in a Murine Model of T. cruzi Infection

For the monotherapy in vivo trial, the FDA drugs and benznidazole were tested at the same dose (100 mg/kg) (Figure 8). The results showed that Olmesartan, Zafirlukast, and Tadalafil reduced parasitemia by 47.32, 43.67, and 37.05%, respectively, after 8 h, although it is not on par with benznidazole (79.17%); the Kruskal–Wallis test indicates that the FDA-approved drugs did not exhibit the same activity as Bzn (*p* < 0.05), whilst Zafirlukast and Olmesartan exhibited the best activity. It should be noted that Bzn was selected as a control to compare the evaluated compounds against the most widely used therapeutic standard; this approach is consistent with preclinical evaluation strategies that use the current standard treatment as a reference point. These findings suggest that the three FDA drugs retain their trypanocidal activity in an in vivo model; therefore, they could be presented as potential repositioning candidates to treat Chagas disease. The known pharmacological properties of these compounds could be related to the observed effects; however, such associations should be interpreted with caution. Zafirlukast, a leukotriene modulator, can modulate the inflammation induced by the parasite; Olmesartan, an angiotensin II receptor antagonist, could reduce the inflammation and oxidative stress, which could present a cardioprotective effect, which is of most importance for this disease since one of the most affected organs is the heart, so in this way the tissue damage associated with the infection could be reduced; finally, Tadalafil, a phosphodiesterase type 5 inhibitor, could have some influence on nitric oxide signaling and promote indirect antiparasitic effects. Nevertheless, the extent to which these mechanisms contribute to the trypanocidal activity observed in this study remains to be elucidated.

Although the efficacy observed in short-term treatments, such as the one performed here, may not reflect a sustained or cumulative effect, this is why long-term trials are required to evaluate the total parasite reduction and the possibility of parasitological cure.

Finally, combining these compounds with the reference drug (Bzn) could enhance its efficacy and reduce the toxicity of the latter, as seen in other studies [6,7]. All this together reinforces the idea that drug repositioning does represent a promising strategy to improve therapies already available for treating Chagas disease, while highlighting the need for further mechanistic and long-term studies.

### 3.6. Molecular Dynamics Studies of FDA Drugs Used In Vivo Trial

In drug discovery and drug repurposing, MD studies are described as powerful for refining the docking results and help to explain more in depth the behavior of each ligand in complex with protein target [23]. In this regard, the *Tc*TS protein has not been crystallized to date; therefore, a model was generated for in silico studies. This model proved to be stable during MD simulations (RMSD = 2.5 Å, RMSF < 1.7 Å), indicating that the protein is suitable for evaluating stability in both its free form and when complexed with a ligand. Based on in vivo results, the drugs zafirlukast and tadalafil were selected for MD studies on the active site of *Tc*TS for a total of 120 ns. Olmersatan was selected for simulation at the predicted allosteric site. In addition, to compare the reference inhibitors ZAE35 and Z109494586, they were subjected to MDS in each site of interest. Previous studies have shown that this simulation time is suitable to assess the stability of a complex of interest and to gain insight into its potential behavior in a biological system [24,25].

In general, RMSD, RMSF, and Rgyr analyses carried out for all trajectories showed acceptable stability. Specifically, olmesartan exhibited a high fluctuation during the first 25 ns. However, it then reached stability. This suggests that during MD, the ligand accommodates itself in a more favorable orientation, which differs from that observed during docking (semi-rigid). Our findings are consistent with the main in vivo data. In addition, the results highlight the importance of MD simulations for refining rigid methods such as docking and for proposing potential mechanisms of inhibition for the *T. cruzi* TS target protein. Further studies could focus on obtaining the *Tc*TS crystal structure in complex with the drugs mentioned in this study to confirm their binding mode within the cavity of interest, thereby advancing the design of *T. cruzi* TS inhibitors as potential antiparasitic agents.

## 4. Materials and Methods

### 4.1. Modeling of T. cruzi Trypanothione Synthetase and Its Preparation for Molecular Docking

To perform the 3D modeling, the TS amino acid sequence from *T. cruzi* CLBrener strain with code AAG15409.1 [26] was downloaded from the NCBI protein database and entered into the modeling software SwissModel [27]; the *Leishmania major* TS with code PDB: 2VOB [18] was used as a scaffold. The structure obtained was analyzed to obtain the Ramachandran plot to determine the quality of the modeling. Using the open-access software UCSF-Chimera 1.16 [28], the hydrogens and charges were added to the structure obtained with the Dock Prep tool (https://www.cgl.ucsf.edu/chimerax/docs/user/tools/dockprep.html, accessed on 30 November 2025), and finally it was converted to PDBQT format, thus adding the Gasteiger charges with the MGLTools 1.5.7 software.

### 4.2. Ligand Library Preparation

From the BindingDB database [29] the drugs were obtained in a single file in .tsv format, using the Open Babel 3.1 software [30]; all those that were repeated were eliminated. Using the same software, the file was converted to .smi format. Next, each drug was separated into an individual .sdf format file, and finally the molecules were minimized and converted to .mol2 format; the force field applied was MMFF94 [31].

### 4.3. Molecular Docking and Analysis

AutoDock Vina 1.1.2 was used to perform molecular docking. Molecular docking was performed using the few controls that have been reported in the literature to be inhibitors of *Tc*TS [6,22] at the different sites of interest: catalytic site (X = −11.729, Y = −10.560, and Z = −2.080), blind docking site, and probable allosteric site (X = −27.871, Y = −0.318, and Z = −17.397). The size of the box was compared to the largest compound in the library, which was Tannic Acid, thus obtaining a box of 24 × 24 × 24 Å. From these results, the limit of the docking score and the most common interactions of the ligand–protein interaction profile were marked, and a consensus was created for comparison with the ligand–protein interaction profiles of FDA-approved drugs. Molecular docking analysis was performed using two criteria: docking score and ligand–protein interaction profile. Comparison of the docking score of the FDA drugs with the control ligands was performed; from this comparison, those drugs that were very close or had better docking scores were chosen, and, in this way, the top 20 drugs were selected for each of the sites mentioned above.

For the profile of ligand–protein interactions, the comparison was also made against the controls, selecting those residues that appear more frequently in the mentioned consensus, which can be observed in Figure 3, Figure 4 and Figure 5, mentioned as PS (percentage of similarity). In this way, the FDA drugs were selected based on those with the highest percentage of similarity.

### 4.4. In Vitro Trypanocidal Assay on Blood Trypomastigotes

The assay was performed following the methodology previously reported [21]. The parasites were collected from female mice of the CD1 strain, weighing 25–35 g, infected intraperitoneally with 1 × 10^5^ blood trypomastigotes. Two groups were formed: one with the NINOA strain and the other with the INC-5 strain of *T. cruzi*. After reaching the peak of parasitemia (approximately 2–4 weeks), infected blood was withdrawn by cardiac puncture, using sodium heparin as an anticoagulant agent. Subsequently, the blood was adjusted to a concentration of 1 × 10^6^ trypomastigotes/mL by diluting in physiological saline.

First, a screening was performed to identify if the drugs had trypanocidal activity. The drugs were acquired from Sigma-Aldrich^®^ (Toluca, Mexico). In a 96-well microplate, 90 µL of the blood containing parasites and 10 µL of the selected FDA drugs were added to a final concentration of 12.5 µg/mL. The reference drugs, Nfx and Bzn, were used as positive controls at the same concentration, while the vehicle in which the drugs were dissolved (DMSO 1%) was used as a negative control. Each compound, as well as the controls, was tested in triplicate. The microplate was incubated at 4 °C for 24 h, after which the remaining trypomastigotes were counted using the modified Pizzi-Brenner method [32]. This procedure consisted of taking 5 µL of blood from each well, depositing it on a slide, covering it with an 18 × 18 mm coverslip, and then counting 15 random fields under a brightfield optical microscope at 40X magnification. The percentage of lysis for each compound was calculated by subtracting the number of live trypomastigotes observed in the drug treatment wells from the number of live trypomastigotes in the negative control (no drug). Those compounds whose percentage of lysis was equal to or higher than the reference drugs (Nfx and Bzn) were selected to continue with the determination of the LC_50_. For this purpose, the compounds were evaluated at five different concentrations by serial dilutions (100–6.25 µg/mL), determining the percentage of lysis and calculating the LC_50_ using the Probit method. Finally, the results were converted into micromolar units.

### 4.5. Short-Term In Vivo Assay

The methodology used was previously reported [31]. For the short-term in vivo monotherapy trial, female mice of the CD1 strain weighing between 30 and 40 g were inoculated intraperitoneally with blood trypomastigotes at a concentration of 1 × 10^5^ trypomastigotes/mL with the NINOA strain. After the necessary days to reach parasitemia (12–18 dpi), the mice were separated into 5 groups, each group containing three mice; the first group was administered only 4% gum arabic, which was the vehicle for the drugs, the second group with Bzn, the third group with Tadalafil, the fourth group with Olmesartan and the fifth with Zafirlukast; all were administered at a dose of 100 mg/kg. The time of administration was counted as zero hour; 5 µL of blood was taken from the caudal vein of each mouse. A trypomastigote count was performed following the Pizzi-Brenner method described above, and the procedure was repeated at 2, 4, 6, and 8 h. The percentage of lysis was calculated from the comparison of the number of trypomastigotes counted at zero hour for each of the groups.

### 4.6. Molecular Dynamics Simulations (MDS)

Molecular dynamics (MD) simulations were performed using GROMACS version 2024 for 120 ns at a temperature of 300 K. The topology of each compound was generated with the ACPYPE Antechamber module with the General Amber Force Field as previously described by Wang et al. [33]. The system was solvated by adding water molecules to a dodecahedral box, employing the TIP3P water model. Subsequently, sodium and chlorine ions were added to neutralize the system, followed by energy minimization using the steepest descent algorithm for 50,000 steps. Equilibration was carried out at 300 K in two stages: first under NVT conditions (constant number of particles, volume, and temperature) using a V-rescale thermostat with a time constant (tau_t) of 0.1 ps to assign velocities according to the Maxwell–Boltzmann distribution, and second under NPT conditions (constant number of particles, pressure at 1 atm, and temperature) using the same thermostat along with a Berendsen barostat with time constants (tau_t and tau_p) of 0.1 and 2.0 ps, respectively. Lastly, root mean square deviation (RMSD), root mean square fluctuation (RMSF), and radius of gyration were used to analyze the stability of each complex [34].

## 5. Conclusions

In this study, a molecular docking-based virtual screening at three different sites of trypanothione synthetase allowed the identification of different FDA drugs as potential trypanocides. Tadalafil, Zafirlukast, and Olmesartan showed similar or better LC_50_ than the reference drugs in an in vitro model, and retain their trypanocidal activity in an in vivo model, reducing the parasitemia approximately by 50% after 8 h. Although these drugs show trypanocidal activity, it is still necessary to demonstrate that they act through inhibition of the *Tc*TS enzyme or some other mode of action.

## Figures and Tables

**Table 1:** Docking score (DS) of the top 20 FDA drugs and control inhibitors on the three sites of *Tc*TS. Compounds are identified by numerical labels (1–20) for clarity and ease of comparison with the corresponding figures.

Catalytic Site		Potential Allosteric Site		Blind Docking—FDA Drugs	
Drug	DS (kcal/mol)	Drug	DS (kcal/mol)	Drug	DS (kcal/mol)
1. Etoposide	−9.4	1. Itraconazole	−9.4	1. Dutasteride	−10.9
2. Telbivudine	−9.4	2. Ergotamine	−9.3	2. Tipranavir	−10.5
3. Nilotinib	−9.2	3. Dutasteride	−9.2	3. Ergotamine	−10.5
4. Alendronate	−9.1	4. Argatroban	−9.1	4. Aprepitant	−10.4
5. Eptifibatide	−9.1	5. Adapaleno	−9.1	5. Irinotecan	−10.3
6. Irinotecan	−9	6. Nilotinib	−9.1	6. Lomitapide	−10.3
7. Regorafenib	−8.9	7. Flucytosine	−9	7. Dactinomycin	−10.2
8. Dihydroergotamine	−8.8	8. Olmesartan	−8.9	8. Pimozide	−10.1
9. Telithromycin	−8.8	9. Irinotecan	−8.9	9. Eltrombopag olamine	−10.1
10. Raltegravir	−8.8	10. Paliperidone	−8.9	10. Dihydroergotamine	−10
11. Zafirlukast	−8.7	11. Propoxycaine	−8.9	11. Digoxin	−10
12. Tadalafil	−8.7	12. Oxytetracycline	−8.9	12. Ponatinib	−10
13. Cyanocobalamin	−8.7	13. Telbivudine	−8.9	13. Lurasidone	−10
14. Lomitapide	−8.7	14. Conivaptan	−8.9	14. Conivaptan	−10
15. Sorafenib	−8.7	15. Tadalafil	−8.8	15. Clofazimine	−9.9
16. Ergotamine	−8.6	16. Sulfinpirazona	−8.8	16. Telmisartan	−9.9
17. Glyburide	−8.6	17. Oxymorphone	−8.8	17. Regorafenib	−9.9
18. Linagliptin	−8.6	18. Rifaximina	−8.8	18. Zafirlukast	−9.8
19. Apixaban	−8.6	19. Idelalisib	−8.8	19. Bexarotene	−9.8
20. Hydroxyzine	−8.6	20. Elvitegravir	−8.7	20. Digitoxin	−9.8
ZEA35	−8.7	Z109494586	−9	Z363062290	−9.7

**Table 2:** Percentage lysis and LC_50_ values of the four FDA drugs on *T. cruzi* strains NINOA and INC-5.

Drug	% Lysis at 12.5 µg/mL		LC_50_ µM	
	NINOA	INC-5	NINOA	INC-5
Tadalafil	33 ± 6	24 ± 11	160 ± 10	134 ± 7
Zafirlukast	47 ± 3	36 ± 9	32 ± 3	30 ± 4
Olmesartan	29 ± 8	42 ± 2	78 ± 5	52 ± 4
Raltegravir	45	27 ± 7	92 ± 6	177 ± 9
Nifurtimox	29 ± 5	25 ± 4	161 ± 33	255 ± 39
Benznidazole	32 ± 6	30 ± 6	220 ± 40	337 ± 34

## Data Availability

Data Availability Statements are available in the manuscript.
